# A Notch signaling pathway-related gene signature: Characterizing the immune microenvironment and predicting prognosis in hepatocellular carcinoma

**DOI:** 10.1515/jtim-2024-0020

**Published:** 2025-01-10

**Authors:** Qingmiao Shi, Shuwen Jiang, Yifan Zeng, Xin Yuan, Yaqi Zhang, Qingfei Chu, Chen Xue, Lanjuan Li

**Affiliations:** State Key Laboratory for Diagnosis and Treatment of Infectious Diseases, National Clinical Research Center for Infectious Diseases, National Medical Center for Infectious Diseases, Collaborative Innovation Center for Diagnosis and Treatment of Infectious Diseases, The First Affiliated Hospital, Zhejiang University School of Medicine, Hangzhou 310003, Zhejiang Province, China

**Keywords:** hepatocellular carcinoma, Notch signaling pathway, risk model, immune microenvironment, prognosis

## Abstract

**Background and Objectives:**

Prior studies have highlighted an escalating global burden of hepatocellular carcinoma (HCC). The Notch signaling pathway regulates the initiation and development of HCC and determines the HCC prognosis.

**Methods:**

The expression data of genes related to the Notch signaling pathway were acquired from public databases. To filter prognostic gene signatures and establish the risk model, the analyses of consensus clustering, least absolute shrinkage and selection operator (LASSO), and multivariate Cox were conducted. Subsequently, the risk stratification was optimized using a decision tree and nomogram. The immune landscapes were revealed utilizing the single-sample gene set enrichment analysis, and tumor immune dysfunction and exclusion score.

**Results:**

According to the mRNA expression profile of Notch signaling pathway-related genes, HCC patients were stratified to three clusters, which have different survival probability and immune infiltration characteristic. Subsequently, we developed a risk model based on five prognostic Notch signaling-related gene signatures (SPP1, SMG5, HMMR, PLOD2, and CFHR4). The model demonstrated an accurate estimation of overall survival, revealing alterations in immune status and immunotherapy sensitivity among HCC patients with different risk scores.

**Conclusions:**

This study constructed a Notch signaling pathway-related prognostic model, offering valuable insights for the assessment of immune characteristics and immunotherapy responses in HCC patients.

## Introduction

As per Global Cancer Statistics 2023, liver cancer ranks as the sixth most prevalent cancer, witnessing 900,000 new cases.^[1]^ Additionally, it stands as the 3^rd^ predominant cause of worldwide cancer-related mortality, accounting for 830,000 annual fatalities.^[2]^ Meanwhile, a persistent rise is observed in both the incidence and mortality rates of liver cancer.^[3,4]^ Of all liver cancer cases, 80% are attributed to hepatocellular carcinoma (HCC). Most patients with HCC are diagnosed with tumors at an advanced stage, often accompanied by cirrhosis.^[5,6]^ Therefore, only 20%–30% of patients qualify for surgical therapy, which is considered the optimal treatment modality for HCC.^[7]^ Since 2017, immunotherapy has established itself as a breakthrough therapeutic approach for advanced-stage HCC.^[8]^ In 2020, an IMbrave150 phase III trial reported that atezolizumab combined with bevacizumab as a first-line therapy exhibited more favorable clinical outcomes than those of sorafenib therapy.^[9,10]^ Moreover, the therapeutic efficacy of tremelimumab plus durvalumab as a first-line treatment surpassed that of sorafenib.^[11]^ Despite advancements in systemic treatment strategies, accurate diagnostic and prognostic biomarkers for early-stage HCC remain elusive. Hence, it is pertinent to discover reliable biomarkers for the diagnosis and prognosis of HCC.

The heterozygous deletion of Notch was identified on the X chromosome of Drosophila.^[12]^ Notch receptor is a type of heterodimeric cell membrane protein.^[13]^ In humans, four Notch receptors have been identified: NOTCH1, NOTCH2, NOTCH3, and NOTCH4. These receptors interact with transmembrane ligands, including Delta-like (DLL1, DLL3, and DLL4) and Jagged/Serrate (JAG1 and JAG2) family proteins, on neighboring cells to transduce signals.^[14]^ The activated Notch signaling pathway triggers the classical Notch target genes, encoding the HES, MYC, and P21 family members.^[15]^ The Notch signaling pathway regulates organism development and homeostasis.^[16]^ Notably, mutations in Notch signaling pathway-related proteins have been strongly associated with several conditions, such as familial congenital scoliosis and Alagille syndrome.^[17,18,19]^ The aberrant Notch signaling activation may induce nonalcoholic fatty liver disease, while prolonged deficiency of this pathway can lead to cartilaginous homeostasis imbalance and bone destruction.^[20,21]^ Additionally, the dysregulation observed in the Notch signaling causes various pulmonary disorders.^[22,23,24,25]^ Thus, Notch signaling dysregulation markedly promotes the onset and progression of congenital or nongenetic diseases.

The Notch signaling pathway exerts an effect on the onset and progression of HCC. The gain and loss of the function of Notch may contribute to the HCC tumorigenesis. For example, the overexpression of endogenous NOTCH1 may inhibit the signal transduction of β-catenin, promoting the epithelial-to-mesenchymal transition and enhancing the invasive and migratory ability of HCC. Ankur Sharma *et al*. reported the upregulation of NOTCH2, DLL4, and HES1 in HCC samples.^[26]^ Sarah Luiken *et al*. revealed that the Notch target gene HES5 exerts tumor-suppressive effects by inhibiting HES1 and downregulating the pro-proliferative MYC target genes, such as ODC1 and LDHA.^[27]^ In contrast, HES5 promoted oncogenesis by disrupting the formation of AKT-dependent liver cancer. Previous studies have reported that the downstream signaling mediators of Notch (DLL4 and JAG1) and the JAG1/NOTCH2 signaling pathway inhibit the progression of HCC.^[28]^

In this study, Notch signaling pathway-related genes were comprehensively examined using bioinformatic analysis to identify significant prognostic genes for HCC. Next, a prognostic model was established on the basis of the mRNA expression levels of genes related to the Notch signaling pathway. This model aims to facilitate the development of clinical treatment and prognostic assessment strategies for HCC patients.

## Materials and methods

### Data selection and processing

The Cancer Genome Atlas (TCGA) database was searched to acquire the genomic mutation atlas, raw mRNA expression profiles, and related clinical information about liver hepatic cellular carcinoma (LIHC) (*n* = 421). In addition, the clinical records and the RNA sequencing (RNA-seq) data of 203 individuals with HCC were retrieved by the HCC Database 18 (HCCDB18) dataset.^[29]^ The Gene Expression Omnibus (GEO) database was utilized to retrieve the GSE14520 and GSE76427 datasets, comprising clinical survival information and high-throughput mRNA expression information. Additionally, 47 genes related to the Notch signaling pathway were extracted from the Molecular Signatures Database (MSigDB).^[30]^ A list of these 47 genes is shown in Table S1 (supplementary materials).

Exclusion criteria was applied to remove samples lacking mRNA expression profiles and clinical data. Subsequently, the Ensembl ID was matched to the gene symbol, and the mean value of gene expression was employed for further analysis.

### Consensus clustering

The “ConsensusClusterPlus” package was employed to identify the clusters of HCC samples with 15 Notch signaling pathway-related genes significantly associated with prognosis. The consensus cumulative distribution function (CDF) curve was plotted, and the optimal clustering number was assessed on the basis of the delta area plot. When the CDF reaches a proximate ultimate value, the clustering classification results gain the highest credibility, with the corresponding k value indicating the optimum value of k. The delta area plot illustrates the relative change in the area under the CDF curve at “k” and “k–1”. Principal component analysis (PCA) was performed to further verify the rationality of the identified clusters. The heatmaps were utilized to assess the correlation between the gene expression levels and the prognosis of HCC patients.

### Differentially expressed gene analysis and functional enrichment assessment

Differential expression analysis of the mRNA expression profile was performed to identify differentially expressed genes (DEGs) between HCC and adjacent control samples from TCGA. The analysis was performed utilizing the R package “limma” based on the following criteria: false discovery rate (FDR) < 0.05; |log_2_ (fold-change (FC))| > log_2_(1.5). Volcano plots were utilized to display the DEGs between two clusters. In these plots, red dots represented upregulated DEGs, blue indicated downregulated, and gray dots represented non-significant DEGs. Next, a Venn diagram was used to screen overlapping DEGs among different clusters with the R package “VennDiagrams.” Additionally, the DEGs underwent Gene Ontology (GO) analyses to determine the most significantly enriched biological functions in terms of biological process, cellular component, and molecular function *via* WebGestaltR (V0.4.4). To identify the enriched pathways, the DEGs underwent Kyoto Encyclopedia of Genes and Genomes (KEGG) analysis.^[31]^

### Construction of the risk model

Univariate Cox analysis was carried out to identify the prognostic DEGs. The least absolute shrinkage and selection operator (LASSO) analysis, facilitated by the R package “glmnet”, was employed to narrow down the number of genes in the final model.^[32]^ The optimal coefficient (λ) was 0.0561. Furthermore, the genes identified through LASSO underwent multivariate Cox analysis for the identification of the final set of prognosis related genes. The formula for the risk score calculation is:

$$\begin{array}{c} \text{risk score}=\sum \beta i\times E\chi pi, \end{array}$$

where i indicates prognostic genes, Expi indicates the mRNA expression level of gene *i*, and *β* indicates the regression coefficient of corresponding genes. The surv_cutpoint function of the R package “survminer” was employed to filter the optimal threshold, categorizing the samples into high-risk and low-risk groups.

### Performance assessment of the risk model

The Kaplan-Meier (K-M) analysis and log-rank test were conducted to estimate the overall survival (OS) probability in both risk groups.^[33]^ The receiver operating characteristic (ROC) curve is indicative of the classification impact, while the area under the curve (AUC) can demonstrate the efficacy and sensitivity of the model. Therefore, the AUC and ROC curve were employed to verify the universality of the risk-scoring system in the validation datasets. The risk score distribution in various clusters was then analyzed. Furthermore, the correlation between clinical pathological characteristics (T stage, stage, and grade) and risk score was investigated using analysis of variance (ANOVA).

### Single-sample gene set enrichment analysis

The R package “GSVA” was used to calculate the enrichment scores of Notch signaling signature in tumor and adjacent tissues. Besides, ssGSEA of 28 immune gene sets was performed using genes associated with various types of immunocytes, pathways, functions, and checkpoints.^[34]^ The enrichment scores of immune cells were calculated utilizing the ssGSEA algorithm implemented with the R package “GSVA”. The Notch signaling signature-derived ssGSEA score indicates the proportion of various immunocyte types and immune-related pathways.

### Assessment of drug sensitivity and immunotherapeutic response

Tumor immune dysfunction and exclusion (TIDE) serves as a foundation for identifying the underlying immune checkpoint blockade and establishing biomarkers to predict the immunotherapeutic responses.^[35]^ A low TIDE score indicates a decreased likelihood of immune evasion, suggesting that patients can benefit from immunotherapy. The RNA-seq data of individuals with HCC who underwent immunotherapy were obtained from the GSE135222, GSE78220, and GSE91061 datasets. The sensitivity to antitumor agents was assessed by calculating the half-maximal inhibitory concentration (IC50) value of chemotherapeutic drugs utilizing the R package “pRRophetic”. An analysis was conducted to assess how the high- and low-risk groups differentially responded to antitumor drugs.

### Construction of decision tree and nomogram

A decision tree is a machine learning algorithm for classification and prediction modeling. It provides different decision paths by constructing a tree to visually represent data and facilitate predictions. This study constructed a decision tree to sort subgroups based on age, gender, grade, M stage, N stage, stage, and risk type. The nomogram, a graphical tool based on the regression model, quantifies event risks using diverse prediction factors. This study constructed the nomograms for the prediction of clinical outcomes for HCC patients. The calibration and decision curves were generated to evaluate the performance and sensitivity of the model.

### Cell cultivation

The healthy hepatocyte line (LO2 cells) and human liver cancer cell line (HepG2 cells) were procured from the Chinese Academy of Sciences (Shanghai, China). These cells underwent cultivation in Dulbecco’s modified Eagle medium (Gibco, USA), enriched with 1% penicillin streptomycin (Beyotime, China) and 10% fetal bovine serum (Gibco, USA) at 37°C in an incubator with 5% CO_2_.

### Single-cell RNA sequencing (scRNA-seq) analysis

The GSE125449 dataset was utilized to retrieve scRNA-seq data of HCC samples. The data were filtered based on the following criteria: each gene should be expressed in at least three cells with a minimum of 200 genes per cell.^[36]^ Additionally, the calculation of rRNA and mitochondrial proportion was performed using the Percent-ageFeatureSet function to ensure that the number of genes was < 6000 and the per-centage of mitochondria was < 15%. The unique molecular identifier of every cell was required to be greater than 200. The data underwent standardization and highly variable genes were sorted separately utilizing the FindVariableFeatures function and log-normalization. The ScaleData function was employed to scale all the genes. PCA was used to reduce the dimension with the specified value of 10 for selecting anchor points. Batch correlation was performed using Harmony. The resolution parameter was set to 0.04 with the FindClusters function, dividing cells into 6 clusters. The score of each pathway was assessed and the differential scores for the KEGG_ NOTCH_SIGNALING_PATHWAY in different cells were estimated.

### RNA extraction and quantitative real-time polymerase chain reaction

The RNA easy mini kit (QIAGEN, USA) was utilized to extract total RNA from cell homogenates. The reverse transcription of isolated RNA into complementary DNA (cDNA) was conducted through the PrimeScript RT Master kit (Takara Bio, Japan). The whole process was conducted on ice to suppress RNA degradation. Table S2 contains the list of primer sequences. TB Green Premix (Takara Bio, Japan) was utilized to amplify cDNA, whereas the 2-ΔΔCT method was employed to assess the relative mRNA expression levels. Glyceraldehyde-3-phosphate dehydrogenase (GAPDH) was employed as the internal reference gene.

### Statistical analyses

The R program (version 4.1.3) was utilized to perform all the statistical analyses. LASSO and Cox analyses (univariate and multivariate) were executed to investigate the genes related to prognosis. Comparison of the OS curves of the high- and low-risk groups was carried out by employing log-rank test and K-M analysis. The AUC and ROC analysis were employed to examine the accuracy of the risk model. Significant variations were depicted at *P* < 0.05.

## Results

### Mutation and expression landscapes of Notch signaling pathway-related genes in HCC

**Figure 1:**
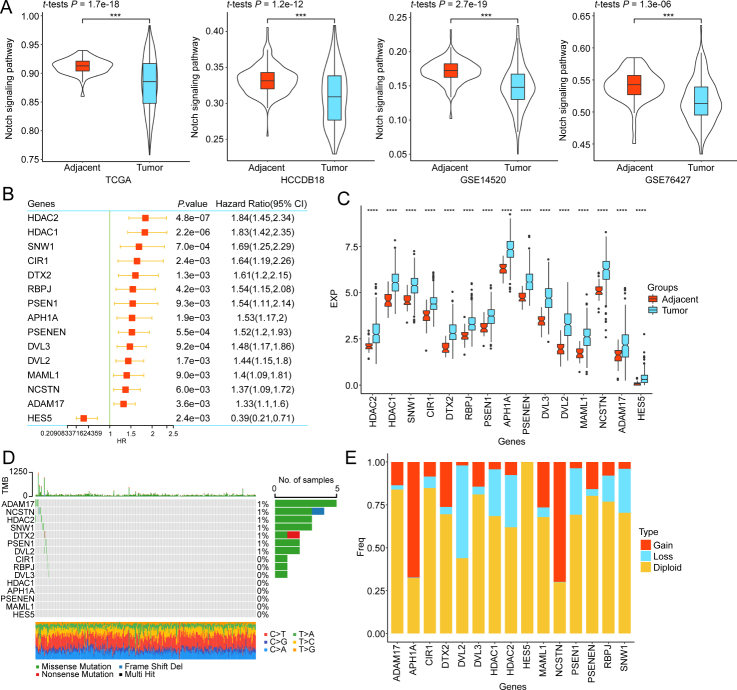
Mutation and expression landscape of Notch signaling-related genes in hepatocellular carcinoma (HCC). Differential scores in single-sample gene set enrichment analysis (ssGSEA) of Notch signaling pathway between tumor and adjacent tissues from four datasets (A). Univariate Cox regression of vital genes in The Cancer Genome Atlas (TCGA)-LIHC cohort (B). Comparative analysis of the expression levels of Notch signaling-related genes between adjacent control tissues and tumor tissues (C). The mutational frequency of Notch-signaling-related genes in HCC (D). The frequency of copy number variation (CNV) in Notch signaling pathway-related genes (E). ^***^P < 0.001, and ^****^P < 0.0001.

This study preliminarily analyzed the differential Notch signaling signature-derived ssGSEA scores between tumor and adjacent tissues in TCGA, HCCDB18, GSE14520, and GSE76427 datasets (Figure 1A). The Notch signaling score of HCC tissue was lower than that of adjacent tissue, indicating the involvement of the Notch signaling pathway in the HCC progression. Univariate Cox regression analysis revealed 15 genes significantly correlated with HCC prognosis (Figure 1B). In HCC tissues, the expression levels of both protective and risk genes were upregulated when compared to adjacent tissues (Figure 1C). The mutation rates in these genes were observed to be less than 1% (Figure 1D). Analysis of the variation in copy numbers revealed that APH1A and NCSTN exhibited increased copy numbers, whereas DVL2, HDAC1, HDAC2, PSEN1, and SNW1 exhibited decreased copy numbers (Figure 1E).

### Identification of three different clusters based on Notch signaling pathway-related genes

**Figure 2:**
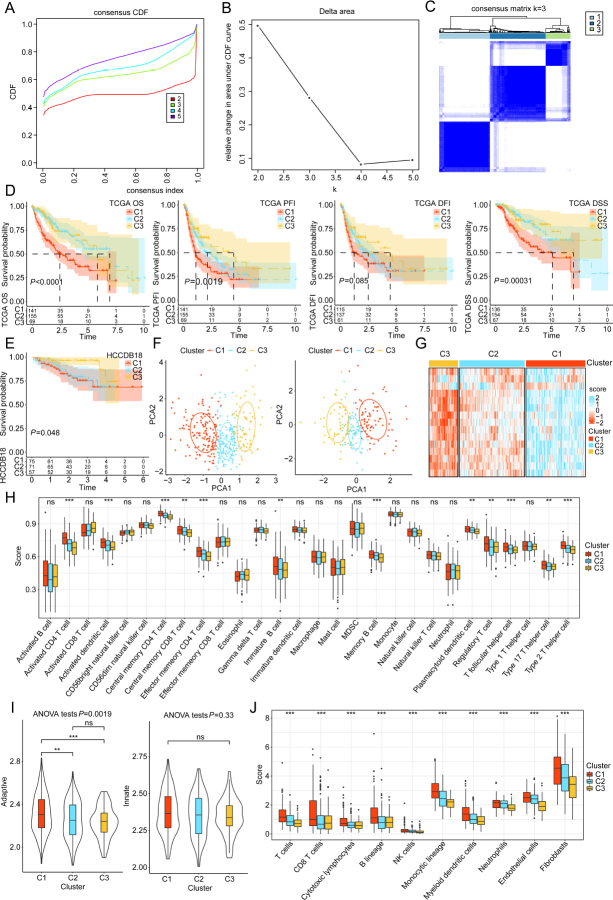
Clusters identification based on Notch signaling pathway-related genes. The cumulative distribution function (CDF) curve and delta area of TCGA cohort (A-B). The consensus heatmap of samples when k = 3 (C). The Kaplan-Meier (K-M) analysis of overall survival (OS), progression-free interval (PFI), disease-free interval (DFI), and disease-specific survival (DSS) in the TCGA cohort (D). The K-M survival curves of the HCCDB18 cohort (E). Principal component analysis (PCA) of the 3 clusters in the TCGA cohort (F). The heatmap of 15 gene expression levels in the three clusters (G). The immune cell infiltration scores of 28 immune cells (H). The adaptive and innate immunity scores among three clusters (I). The distribution of 10 immune cell scores in the three clusters (J). ^**^P < 0.01, and ^***^P < 0.001; ns: non-significant.

Consensus clustering analysis was carried out using the mRNA expression profile of 15 genes related to Notch signaling pathways (Figure 2A and 2B). The rationality and stability of the result were evident in the CDF delta area curve, particularly when HCC patients were stratified into three different clusters (Figure 2C). The K-M curves depicted significant difference in the OS, progression-free interval (PFI), disease-free interval (DFI), and disease-specific survival (DSS) between the three clusters in TCGA cohort (Figure 2D). Notably, the C3 cluster exhibited the most favorable prognosis, and similar survival result was observed in HCCDB18 cohort (Figure 2E). PCA plot revealed the distinct molecular profiles of the three clusters, with minimal differences within the groups but pronounced differences between them (Figure 2F). The heatmap depicted the expression levels of HCC prognosis-related genes across the three clusters. These genes exhibited universal upregulation in the C1 cluster, whereas downregulation was observed in the C3 cluster (Figure 2G).

Further analysis was performed to assess the infiltration levels of immune cells in distinct clusters. Data on infiltrating immune cells were retrieved from previous studies.^[37,38]^ We found that the proportions of activated CD4^+^ T cells, central memory CD4^+^ T cells, effector memory CD4^+^ T cells, and type 2 helper T cells varied across different clusters. The C1 cluster exhibited a high immune score, whereas decreased immune score were observed in C3 (Figure 2H). Additionally, the adaptive immune landscape demonstrated significant differences between C1 and C2, as well as between C1 and C3 (Figure 2I). However, the innate immune landscape showed no substantial variations between the three clusters. ssGSEA was executed to calculate enrichment scores for diverse immune cell subpopulations within the aforementioned clusters. Compared with those in other clusters, the abundances of B cells, CD8^+^ T cells, cytotoxic lymphocytes, endothelial cells, fibroblasts, monocytic lineage, myeloid dendritic cells, natural killer (NK) cells, neutrophils, and T cells were markedly upregulated in the C1 cluster (Figure 2J).

### Analysis of DEGs among different clusters

**Figure 3:**
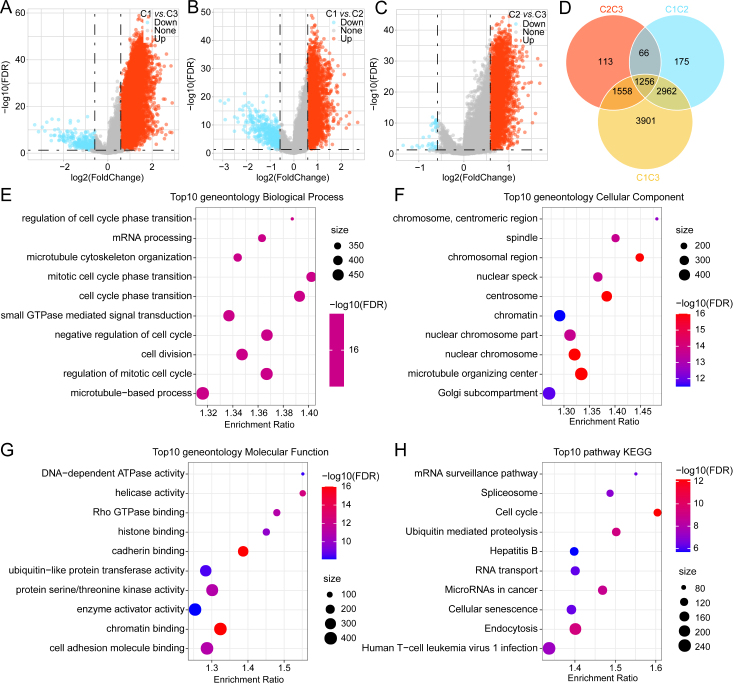
Differentially expressed gene (DEG) analysis across different clusters. Volcano plots depicting DEGs, where red indicates upregulation and blue color signifies downregulation (A-C). Intersection of DEGs between the three clusters (D). Findings of Gene Ontology (GO) enrichment analysis depicting the DEG enrichment in biological process (BP), cellular component (CC), and molecular function (MF) (E-G). The bubble plot illustrating the DEG enrichment in the Kyoto Encyclopedia of Genes and Genomes (KEGG) pathways (H).

Next, this study analyzed the DEGs between the following pairs: C1 and C2; C2 and C3; C1 and C3. Volcano curves were plotted to visualize the DEGs (Figure 3A-3C). Additionally, a Venn diagram was created to illustrate the overlapping DEGs (Figure 3D). Subsequently, the DEGs from the three clusters were subjected to KEGG and GO analyses. In particular, the DEGs underwent enrichment in different GO terms as follows (Figure 3E-3G): biological process: mRNA processing, cell cycle phase transition, and cell division; cellular component: centrosome, nuclear chromosome, and microtubule organizing center; molecular function: DNA-dependent ATPase and chromatin binding.

KEGG pathway analysis depicted that the DEGs were enriched in various pathways, including cell cycle, ubiquitin-mediated proteolysis, and endocytosis (Figure 3H).

### Development and validation of the prognostic model

**Figure 4:**
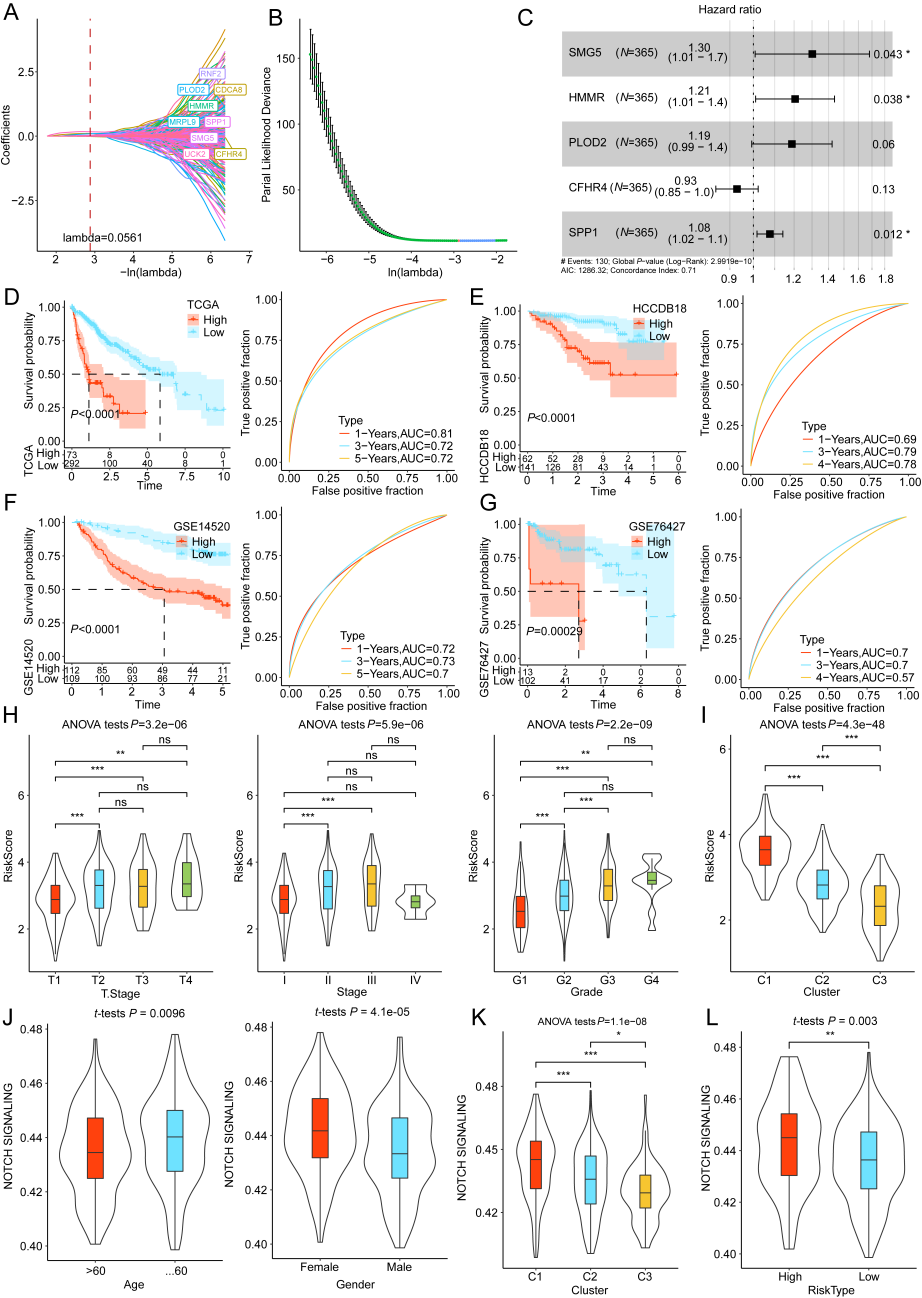
Construction and validation of a risk scoring model in the training group. Nine genes were discovered as the target genes when λ = 0.0561 (A). Screening of optimal parameters (lambda) (B). Stepwise multiple Cox regression analysis revealed five genes, as well as the corresponding coefficients, and demonstrated that they are the optimal gene signature (C). Kaplan-Meier (K-M) curves of both risk patients and the receiver operating characteristic (ROC) curves for 1, 3, and 5-year survival in the (D) TCGA, (E) HCCDB18, (F) GSE14520, and (G) GSE76427 cohorts. (H) The distribution of risk scores in various clinical characteristics of TCGA cohort. The risk scores varied between the clusters (I). The distribution of Notch signaling signature-derived ssGSEA scores in clinical characteristics including age and gender (J). The Notch signaling signature-derived ssGSEA scores varied between the clusters (K). The ssGSEA scores of Notch signaling significantly varied between the high-risk and low-risk groups (L). ^*^P < 0.05, ^**^P < 0.01, and ^***^P < 0.001; ns: non-significant.

To explore the key prognostic genes for HCC, univariate Cox regression was conducted on 1256 DEGs. This analysis identified 854 genes that significantly affected outcomes with HCC patients (*P* < 0.05). LASSO Cox regression was employed to further refine the most critical prognostic gene signature. This analysis identified RNF2, PLOD2, CDCA8, HMMR, MRPL9, SPP1, SMG5, UCK2, and CFHR4 (λ = 0.0561) as the target genes (Figure 4A-4B). Stepwise Cox regression analysis further narrowed down the range and revealed five genes and their corresponding coefficients, suggesting that these five genes constitute the optimal gene signature (Figure 4C).

Following is the formula for the risk score calculation for HCC patients:

risk score = (0.264 × SMG5 expression level) + (0.187 × HMMR expression level) + (0.172 × PLOD2 expression level) – (0.073 X CFHR4 expression level) + (0.074 × SPP1 expression level).

As per the calculated risk score, the patients were classified into high-risk and low-risk groups. K-M analysis depicted that the high-risk group exhibited a reduction in the OS in comparison to the other group. The ROC curves revealed AUC values of 0.81, 0.72, and 0.72 for the prediction of 1-, 3-, and 5-year survival, respectively (Figure 4D). The trend of risk scores observed in the HCCDB18, GSE14520, and GSE76427 cohorts were consistent with those identified in the training cohort (Figure 4E-4G).

Individuals with HCC were categorized on the basis of their clinicopathological features. There was a remarkable variation in risk score distribution between the groups, with an increase in the risk score corresponding to higher T stage, stage, and grade (Figure 4H). Additionally, the risk scores were markedly varied between the three clusters (C1, C2, and C3) (Figure 4I). Subsequently, the distribution of Notch signaling signature-derived ssGSEA score in clinical features was analyzed. The Notch signaling scores varied according to age and gender (Figure 4J). Moreover, the Notch signaling scores varied among C1, C2, and C3 clusters, and also between both risk groups (Figure 4K-L). These findings indicate an enhanced performance of the Notch signaling pathway-related gene signature-based HCC prognostic model.

### Evaluation of tumor microenvironment and treatment strategies for HCC based on risk score

**Figure 5:**
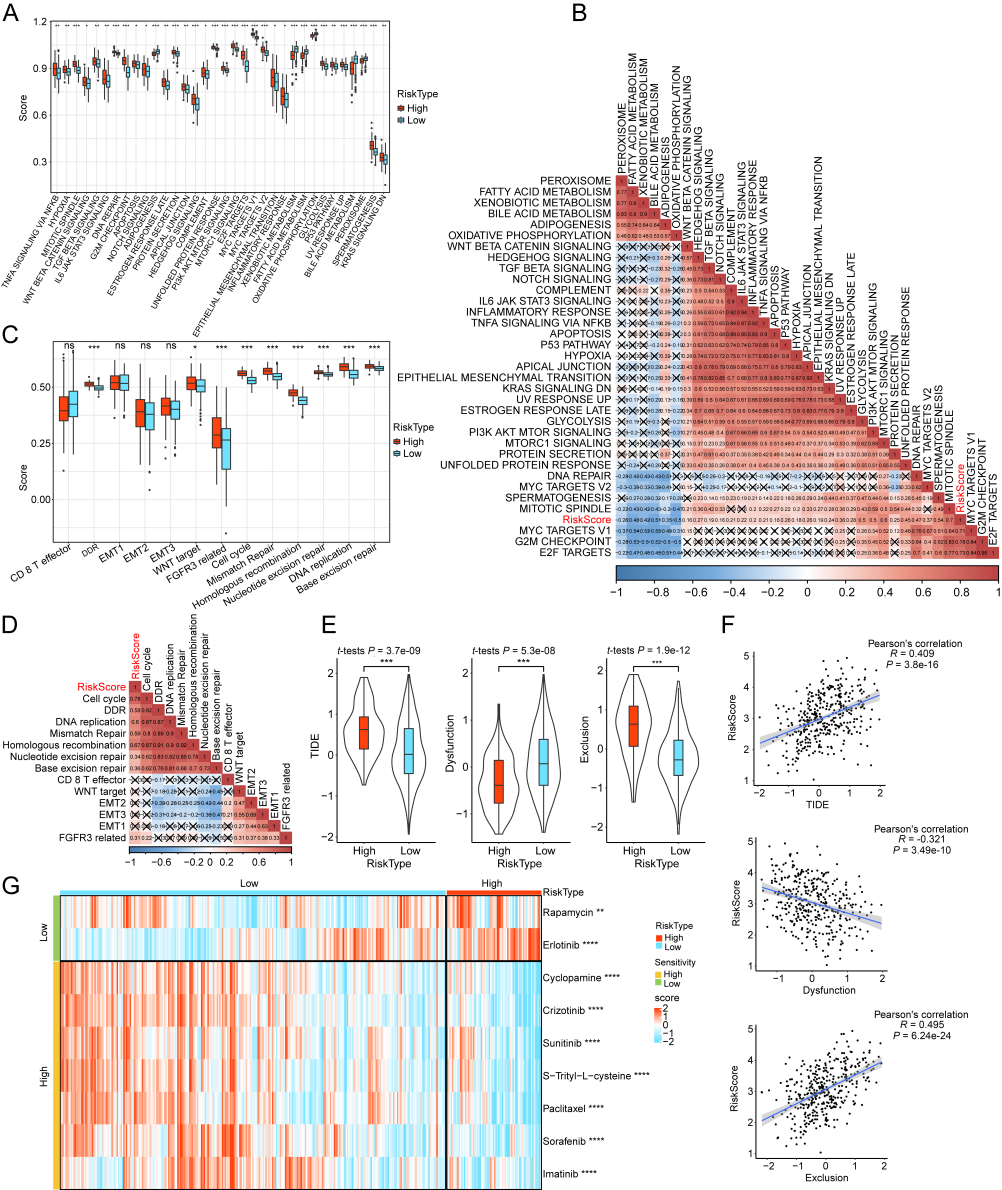
Analysis of pathway, immune score, immunotherapy response, and drug sensitivity in the risk groups. Distribution of 34 functional pathways in the high-risk and low-risk groups within TCGA cohort (A). Examination of the correlation between 34 functional pathways and risk scores (x indicates *P* > 0.05) (B). Assessment of the association between human gene signatures of 13 immunotherapy response-related pathways and risk scores (C). The correlation analysis between the immunotherapy response-related pathways and risk scores (D). The differential tumor immune dysfunction and exclusion (TIDE) scores between the two groups (E). Correlation analysis between risk scores and TIDE/Dysfunction/Exclusion scores (F). Heatmap of differential sensitivities to nine drugs (G). ^*^P < 0.05, ^**^P < 0.01, ^***^P < 0.001, and ^****^P < 0.0001; ns: non-significant.

The correlation between functional pathways and risk score was examined in both high-risk and low-risk groups from the TCGA cohort. The study observed significant differences in the enrichment of 34 pathways, including apoptosis, E2F targets, G2M checkpoint, inflammatory response, IL6-JAK-STAT3 signaling, Notch signaling, TNFA signaling *via* NFKB, and P53 pathway, between patients of the aforementioned groups (Figure 5A). The correlation between 34 functional pathways and risk scores was further investigated. The risk scores were found to be positively correlated with mitotic mTORC1 signaling, PI3K/AKT/mTOR signaling, spindle, glycolysis, and DNA repair (Figure 5B). These pathways are associated with immune response. The human gene signatures of 13 immunotherapy response-related pathways were retrieved from a previous study, which collected samples before programmed death ligand 1 (PD-L1) blockade treatment.^[39]^ In our study, the risk score depicted a positive association with base excision repair, cell cycle, DNA replication, DNA damage response (DDR), homologous recombination, mismatch repair, and nucleotide excision repair (Figure 5C-5D). The low-risk patients, having a low TIDE score, may benefit from immunotherapy. Meanwhile, high-risk patients with heightened exclusion and lower dysfunction scores may exhibit an elevated potential for immune escape. Thus, the limited efficacy of immune checkpoint inhibitor therapy was observed in these patients (Figure 5E). The risk score exhibited a strong correlation with TIDE (*R* = 0.409) and exclusion (*R* = 0.495) scores, and negative correlation with dysfunction (*R* = -0.321) scores (overall *P* < 0.001) (Figure 5F). An assessment of traditional chemotherapy drugs revealed that high-risk patients exhibited sensitivity to cyclopamine, crizotinib, sunitinib, S-trityl-L-cysteine, paclitaxel, sorafenib, and imatinib. Conversely, the low-risk patients exhibited sensitivity to erlotinib and rapamycin (Figure 5G).

### Efficacy of the risk model in immunotherapy datasets

**Figure 6:**
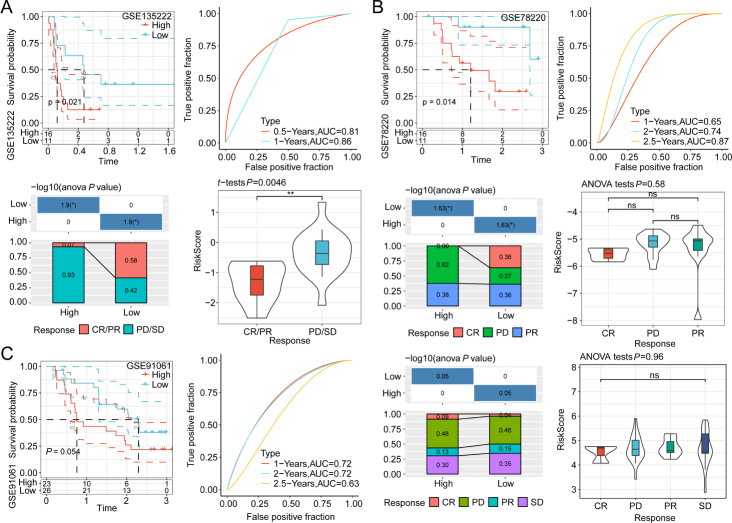
Comparative analysis of the performance of immunotherapies in diverse datasets. The immunotherapy response distribution map, receiver operating characteristic (ROC) curve, and survival curve in the GSE135222 (A), GSE78220 (B), and GSE91061 datasets (C). ^*^P < 0.05, and ^**^P < 0.01; ns: non-significant.

The low-risk group, determined by the risk score, may exhibit an immune-hot subtype within tumors. To further examine the predictive efficacy of risk score for immunotherapy response, the clinical information and transcriptome data of HCC patients treated with immunotherapy were analyzed using the GSE135222 (anti-PD1 therapy), GSE78220 (anti-PD1 therapy), and GSE91061 (anti-CTLA4 and anti-PD1 therapy) datasets. In the GSE135222 dataset, the survival probability substantially varied between the two groups categorized per the median risk score (*P* = 0.021). ROC analysis indicated that the AUC values for the prognosis of 0.5-year and 1-year survival were 0.81 (95% CI = 0.64–0.97) and 0.86 (95% CI = 0.68–1.04), respectively. This suggests that the model showed an excellent performance in patient outcome prediction. The elevated proportion of high-risk patients with progressive disease (PD)/stable disease (SD) was observed than that of the low-risk patients (Figure 6A). This indicated that the benefit of immunotherapy was minimal for high-risk patients. Consistent results were revealed in the TIDE score analysis. Additionally, similar findings were obtained during the examination of GSE78220 (Figure 6B) and GSE91061 (Figure 6C) datasets.

### Development of a decision tree and nomogram based on the risk model

**Figure 7:**
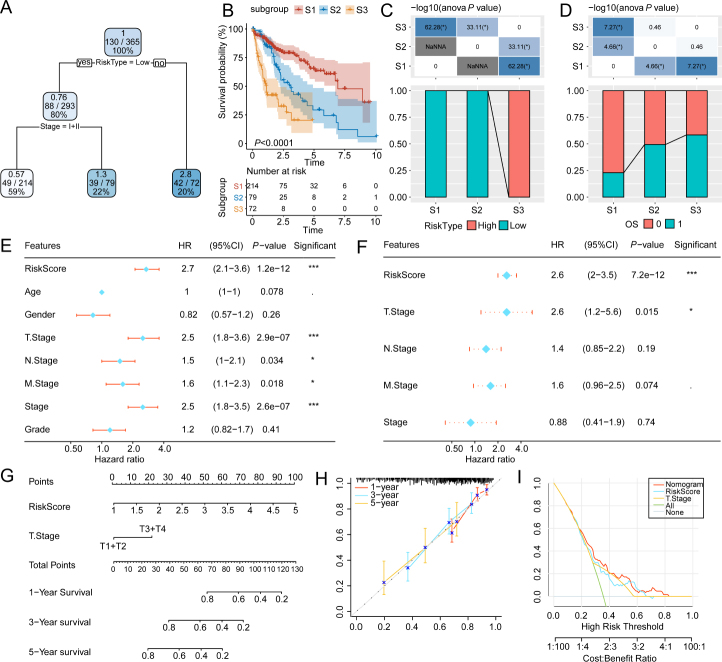
Optimization of the prognostic model and survival prediction by decision tree and nomogram. The construction of a decision tree on the basis of fullscale annotations (A). The differential overall survival between the three risk subgroups of liver hepatocellular carcinoma (LIHC) (B). Comparative analysis of risk scores between different subgroups (C). Comparative analysis of survival status between distinct subgroups (D). Univariate and multivariate Cox regression analyses of clinicopathological features and risk scores (E and F). The nomogram was constructed on the basis of risk scores and other clinicopathological features (G). Comparative analysis of predicted calibration curves and standard curves for 1, 3, and 5-year survival (H). The decision curve analysis (DCA) of nomogram (I). ^*^P < 0.05, and ^***^P < 0.001,

A decision tree was generated by incorporating various factors, including age, gender, grade, stage, M stage, N stage, T stage, and risk type of individuals having HCC in the TCGA-LIHC cohort. However, the two key parameters in the decision tree were risk type and stage (Figure 7A). Utilizing these parameters, three different risk subgroups (S1, S2, and S3) were identified. The OS status markedly varied among the three subgroups (Figure 7B). Patients in the risk subgroup S3 exhibited high-risk scores, whereas those in the S1 and S2 groups exhibited low-risk scores (Figure 7C). Furthermore, the survival status varied between distinct risk subgroups (Figure 7D). Univariate and multivariate Cox analyses marked the risk score as the crucial independent risk factor (hazard ratio (HR) = 2.6, 95% CI = 2–3.5, *P* < 0.001) for the HCC patients’ prognoses (Figure 7E and 7F). A nomogram was generated to quantify the risk estimation and survival status of individuals with HCC, using the combination of risk score and other clinicopathological features. The risk score exerted the highest influence on survival prediction (Figure 7G). The predicted calibration curves for 1-year, 3-year, and 5-year survival closely aligned with the standard curve, suggesting excellent performance exhibited by the nomogram (Figure 7H). Decision curve analysis demonstrated that both the nomogram and risk score outperformed the extreme curve (Figure 7I), confirming that their predictive performance for assessing the survival of HCC patients surpassed that of other clinicopathological features.

### scRNA-seq analysis verifying the robustness of the risk model

**Figure 8:**
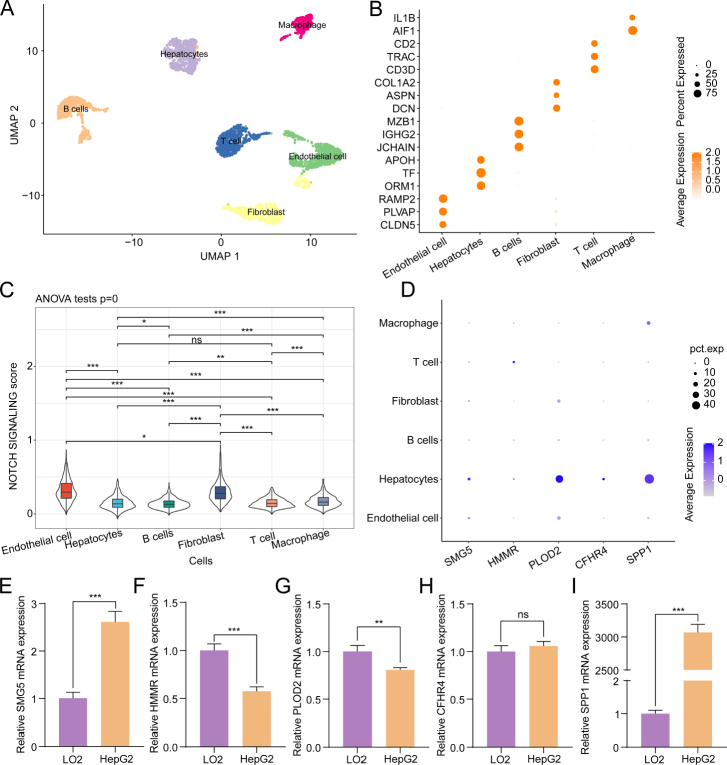
scRNA-seq analysis verifying the robustness of the risk model. Uniform Manifold Approximation and Projection (UMAP) of the GSE125449 dataset (A). The bubble plot depicting upregulated genes in related cell clusters (B). Comparison of the single-sample gene set enrichment analysis (ssGSEA) scores derived from the Notch signaling signature across different clusters (C). The bubble plot depicts the expression of five key risk genes (D). The mRNA expression levels of five risk genes (SMG5, HMMR, PLOD2, CFHR4, and SPP1) in HepG2 and LO2 cells (E-I). ^*^P < 0.05, ^**^P < 0.01, and ^***^P < 0.001; ns: non-significant.

The single-cell transcriptome atlas of HCC from the GSE125449 dataset was analyzed, resulting in the categorization of all cells into the following six clusters: endothelial cells, hepatocytes, B cells, fibroblasts, T cells, and macrophages (Figure 8A). The specific upregulated genes in different cell clusters are shown in the bubble plot (Figure 8B). The pathway score of each cluster was evaluated. The Notch signaling signature-derived ssGSEA score varied between different cells (Figure 8C). Furthermore, the expression of five risk genes was mapped in different cell clusters. PLOD2 and SPP1 underwent upregulation in hepatocytes (Figure 8D). The expression levels of five signature genes in HepG2 cells and LO2 cells were analyzed using qRT-PCR. The mRNA levels of SMG5 and SPP1 in HepG2 cells were elevated as compared to those in the LO2 cells. Meanwhile, the mRNA levels of HMMR and PLOD2 underwent downregulation in HepG2 cells (Figure 8E-8I). The results of the cell-level experiment were consistent with those obtained using the novel prognostic model. These findings indicated the function of the Notch signaling pathway in the occurrence and progression of HCC at the single-cell level and demonstrated the rationality of the developed risk model.

## Discussion

The primary therapeutic modalities for HCC include hepatectomy and liver transplantation. However, traditional clinical signatures cannot effectively predict HCC recurrence due to tumor heterogeneity and consequently do not contribute to the improvement of long-term survival in patients undergoing radical hepatectomy for HCC. Therefore, it is imperative to identify prognostic biomarkers for HCC. Prior research has demonstrated that the Notch signaling pathway regulates the differentiation and proliferation of HCC cells.^[40]^ In this study, 15 Notch signaling pathway-related genes were identified as being associated with HCC prognosis. The expression levels of these 15 genes varied between HCC and adjacent tissues, suggesting their potential involvement in carcinogenesis. Consensus clustering was performed to determine the optimal *k* value. HCC was classified into three molecular clusters, each exhibiting distinct clinical outcomes and immune infiltration statuses. This suggests that the heterogeneous immune status in HCC could serve as a promising prognostic index.

Next, an HCC risk model was established using Notch signaling pathway-related genes. Individuals in the low-risk group depicted an improved OS. Analysis of the immunotherapeutic responses of patients with HCC indicated a substantial variation in the number of patients with PD/SD between the high-risk and low-risk groups in the immunotherapy cohort. This proposes that the risk model has the ability to predict the clinical outcome and immunotherapy response of individuals having HCC. Compared with that of alpha-fetoprotein (AFP), a valuable preoperative and postoperative monitoring index, the performance of the model was favorable for the prediction of HCC clinical outcomes.^[41]^ AFP has been used for over two decades in the screening of HCC. The upregulation of AFP is indicative of a poor prognosis in HCC patients. However, the prognostic application of AFP is limited as it does not provide information on long progression-free survival and OS during treatment.^[42]^ The risk model developed during this research exerts the ability to predict the fate, onset, and progression of HCC throughout the whole process.

The following five prognostic genes were selected using LASSO regression and multivariate Cox regression analyses: SPP1, SMG5, HMMR, PLOD2, and CFHR4. SPP1 was recognized as an immune-related signature associated with unfavorable prognosis in the HCC cohort.^[43]^ Liu *et al*. demonstrated that SPP1 is linked to the formation of the tumor immune barrier by interacting with cancer-associated fibroblasts, thereby restricting immune cell infiltration into the tumor core and influencing immunotherapy efficacy.^[44]^ SMG5, an RNA-binding protein, regulates the oncogenesis and progression of HCC.^[45]^ Additionally, HMMR, a hyaluronic acid receptor, promotes the growth, migration, and differentiation of HCC cells.^[46]^ Prior research reported that endoplasmic reticulum (ER) stress-stimulated HMMR may prolong and attenuate ER stress, providing conducive conditions for the transformation of hepatitis B virus-related cancer.^[47]^ PLOD2, which facilitates the generation of stable collagen cross-links, can activate BIRC3, ultimately enhancing the invasion and migration of HCC.^[48,49,50]^ The study by Yang *et al*. observed a negative correlation between PLOD2 expression and the infiltration of B cells. These outcomes indicate that the risk genes identified in this research can predict the immunotherapy response, progression, and survival outcomes of HCC.

The risk score exhibited a positive correlation with various functional pathways, such as DNA repair, glycolysis, mTORC1 signaling, mitotic spindle, and PI3K/AKT/ mTOR signaling. The PI3K/AKT/mTOR pathway, a classical dysregulated pathway in HCC, promotes treatment resistance and the progression of malignancy in solid cancers.^[51,52]^ mTOR is involved in various cellular processes, including cellular metabolism, immune responses, protein synthesis, and cell death, whereas mTORC1 is known to regulate the Warburg Effect in cancer.^[53]^ In prior research, it was revealed that mTORC1 suppressed NEAT1_2 transcription and paraspeckle biogenesis, thereby upregulating aerobic glycolysis to provide energy for the anabolic activities of HCC cells.^[54]^ These findings provide a foundation for future investigations into the mechanisms through which risk genes can predict HCC prognosis.

The immune landscape of the liver is unique as it contains several distinct immune cells, including Kupffer cells.^[55]^ The maintenance of liver homeostasis is dependent on innate cells and innate cell-like cells, encompassing dendritic cells, gamma delta T cells, granulocytes, macrophages, NK T cells, and NK cells. The immune characteristics and abundance of immune cells varied between the risk groups. Notably, NK cells are one of the key immunoregulatory cells of the innate immune system with cytotoxic ability and can directly kill tumor cells.^[56,57]^ Prior research has indicated that the abundance of tumor-related NK cells is a critical factor in influencing the response to anti-PD1 therapy in HCC.^[58]^ The proportion of immune cells, including NK cells, may serve as an indicator of the immunocompetence and treatment outcomes in HCC.

The sensitivity to traditional chemotherapy drugs varied between the aforementioned groups. The high-risk patients exhibited sensitivity to crizotinib, cyclopamine, imatinib, paclitaxel, sorafenib, S-trityl-L-cysteine, and sunitinib, whereas the low-risk individuals exhibited sensitivity to erlotinib and rapamycin. Consequently, the efficacy of chemotherapy in HCC patients can be determined on the basis of risk score. Particularly, sorafenib, the first molecular targeted drug utilized for unresectable or metastatic HCC treatment, stands out as one of the first-line therapeutic agents in systemic treatments, especially for advanced HCC.^[59]^ Recent studies suggest that the combination of PD-L1 inhibitor atezolizumab and the vascular endothelial growth factor inhibitor bevacizumab demonstrates better overall and progression-free clinical outcomes than those of sorafenib.^[10,60,61]^ PD-L1 inhibitors induce strong immune responses across various cancers. However, their efficacy is observed in only a small subset of patients.^[39]^ The responses to PD-L1 inhibitor treatments are influenced by several factors, such as CD8 T-effector cell phenotype, high neoantigen, and tumor mutation burden. Additionally, the transforming growth factor β signaling in fibroblasts has the potential to suppress immune response by restricting T cell infiltration. These findings identify targets for enhancing the efficacy of PD-L1 inhibitors.

## Conclusion

In conclusion, this study has yielded some valuable results in exploring the Notch signaling pathway in HCC, proposing a risk model based on genes related to the Notch signaling pathway. However, it has not yet been validated for clinical application. Future clinical studies can evaluate the practical application value of this model in guiding the treatment and prognosis assessment of patients with HCC.

### Supplementary Information

Supplementary information is available only at the official site of the journal (www.intern-med.com).

## Supplementary Material

Supplementary Material
