# Supplementary material for: A Notch signaling pathway-related gene signature: Characterizing the immune microenvironment and predicting prognosis in hepatocellular carcinoma
Source: J Transl Int Med. 2025 Jan 10;12(6):553–68. doi: 10.1515/jtim-2024-0020 (PMC12288947; doi:10.1515/jtim-2024-0020)
Supplement: Supplementary file 1 — Supplementary Material [file jtim-2024-0020_sm.pdf]

Supplementary material

A Notch Signaling Pathway-related Gene Signature: Characterizing the Immune Microenvironment and Predicting Prognosis in Hepatocellular Carcinoma

Qingmiao Shi[1†], Shuwen Jiang[1†], Yifan Zeng[1†], Xin Yuan[1], Yaqi Zhang[1], Qingfei Chu[1], Chen Xue[1], Lanjuan Li[1*]

[1]State Key Laboratory for Diagnosis and Treatment of Infectious Diseases, National Clinical Research Center for Infectious Diseases, National Medical Center for Infectious Diseases, Collaborative Innovation Center for Diagnosis and Treatment of Infectious Diseases, The First Affiliated Hospital, Zhejiang University School of Medicine, Hangzhou, 310003, China

[†]These authors contributed equally: Qingmiao Shi, Shuwen Jiang, and Yifan Zeng.

***Corresponding author: Lanjuan Li**

State Key Laboratory for Diagnosis and Treatment of Infectious Diseases, The First Affiliated Hospital, Zhejiang University School of Medicine, No. 79 Qingchun Road, Hangzhou 310003, Zhejiang, China. Tel: +86-0571-87236459; Fax: 86-571-87236459

Email: ljli@zju.edu.cn

Table S1. The gene set of 47 PPAR signaling pathway-related genes.

No.	Gene	No.	Gene	No.	Gene
1	ADAM17	17	DVL3	33	NCSTN
2	APHI1A	18	EP300	34	NOTCH1
3	CIR1	19	HDAC1	35	NOTCH2
4	CREBBP	20	HDAC2	36	NOTCH3
5	CTBP1	21	HES1	37	NOTCH4
6	CTBP2	22	HES5	38	NUMB
7	DLL1	23	JAG1	39	NUMBL
8	DLL3	24	JAG2	40	PSEN1
9	DLL4	25	KAT2A	41	PSEN2
10	DTX1	26	KAT2B	42	PSENEN
11	DTX2	27	LFNG	43	PTCRA
12	DTX3	28	MAML1	44	RBPJ
13	DTX3L	29	MAML2	45	RBPJL
14	DTX4	30	MAML3	46	RFNG
15	DVL1	31	MFNG	47	SNW1
16	DVL2	32	NCOR2		

Note: The gene set was downloaded from the following site: https://www.gsea-msigdb.org/gsea/msigdb/human/geneset/KEGG_NOTCH_SIGNALING_PATHWAY.html?keywords=

Table S2. The information of primers sequences for qRT-PCR.

Primer name	Sequence (5'-3')	Base
SMG5-F	GACGCTTTAACTTTGACACGGAT	23
SMG5-R	GAATTAAAGCAAACACTGGATGAGC	25
HMMR-F	AGGCGGGCGATGAAATGA	18
HMMR-R	CCTGGGTATGAGCAGCACTAC	21
PLOD2-F	GATCATTGCTCCTCTTGTAACTCG	24
PLOD2-R	TTCCATACTCCTACTCTATTCCCTTG	26
CFHR4-F	GGGGATACCATTGAATTTATGTGTA	25
CFHR4-R	TCCACTATGCCTTCCCTACACA	22
SPP1-F	GAAGTTTCGCAGACCTGACATCC	23
SPP1-R	GCTGACTCGTTTCATAACTGTCCTT	25
GAPDH-F	CGCTGAGTACGTCGTGGAGT	20
GAPDH-R	TGCTGATGATCTTGAGGCTGTTG	23
